# Exploratory Machine Learning-Based Classification of Type 2 Diabetes Using Routine Clinical Parameters: A Single-Center Comparative Study

**DOI:** 10.3390/healthcare14121710

**Published:** 2026-06-15

**Authors:** Neşe Bülbül, Rukiye Çiftçi, İpek Atik, Özgür Eken, Nuriye Efe Ertürk, Monira I. Aldhahi

**Affiliations:** 1 Department of Endocrinology and Metabolism, Gaziantep Islam Science and Technology University, Gaziantep 27260, Türkiye; drnesebulbul@yahoo.com.tr; 2 Department of Anatomy, Gaziantep Islam Science and Technology University, Gaziantep 27260, Türkiye; rukiyekelesciftci@hotmail.com; 3 Department of Electrical Electronics Engineering, Gaziantep Islam Science and Technology University, Gaziantep 27260, Türkiye; ipek.atik@gibtu.edu.tr; 4 Department of Physical Education and Sport Teaching, Faculty of Sports Sciences, Inonu University, Malatya 44280, Türkiye; ozgur.eken@inonu.edu.tr; 5 Internal Medicine Nursing Department, Gaziantep Islam Science and Technology University, Gaziantep 27260, Türkiye; nuriye_efe@yahoo.com; 6 Department of Rehabilitation Sciences, College of Health and Rehabilitation Sciences, Princess Nourah bint Abdulrahman University, P.O. Box 84428, Riyadh 11671, Saudi Arabia

**Keywords:** type 2 diabetes mellitus, machine learning, decision tree, random forest, HbA1c, complete blood count, artificial intelligence

## Abstract

**Background/Objectives:** Type 2 diabetes mellitus (T2DM) is a prevalent metabolic disorder associated with substantial long-term morbidity and mortality. Routinely collected anthropometric, biochemical, and hematological variables may contain useful discriminatory information for data-driven classification. This study aimed to compare the apparent classification performance of multiple machine learning algorithms for distinguishing individuals with and without T2DM using routinely obtained clinical parameters in a single-center dataset. **Methods:** This single-center observational study included 160 adults (95 females, 65 males) evaluated at the Endocrinology Outpatient Clinic of Gaziantep Islam Science and Technology University, Faculty of Medicine, Ersin Arslan Training and Research Hospital. The dataset comprised anthropometric measurements, biochemical markers, and complete blood count parameters. SMOTE was applied only within the training folds to address class imbalance and to avoid information leakage. Following fold-internal data preprocessing, which included imputing missing values and feature standardization where appropriate, the dataset was evaluated using stratified 5-fold cross-validation. SHAP analysis was performed to interpret the model predictions. A calibration curve was used to assess the model’s reliability. Eight supervised machine learning models were evaluated with and without HbA1c: Logistic Regression, Linear Discriminant Analysis, Quadratic Discriminant Analysis, Decision Tree, Random Forest, Extra Trees, Gaussian Naive Bayes, and k-Nearest Neighbors. Model performance was evaluated using accuracy, sensitivity, specificity, and F1 score, and ROC curves were used as a diagnostic tool. **Results:** The models were evaluated in two different ways: with and without HbA1c. Random Forest demonstrated the best classification performance in the cross-validated evaluation; without HbA1c, it achieved 92.2% accuracy, 93.9% sensitivity, 97.9% specificity, and a 95.9% F1 score. When HbA1c was included, it achieved 98.0% accuracy, 97.9% sensitivity, 98.8% specificity, and a 99.0% F1 score. Decision Tree and Extra Trees demonstrated strong performance with accuracy rates of 87.6% and 92.8%, respectively, without HbA1c, and 90% and 93.5% when HbA1c was included; in contrast, KNN yielded the lowest accuracy rate (70.6%). Overall, tree-based models performed better than linear classifiers on this dataset. **Conclusions:** Machine learning models based on routine clinical and anthropometric variables demonstrated promising performance for T2DM classification in this single-center dataset; tree-based approaches yielded the most promising results. Including HbA1c improved the models’ ability to classify individuals with and without T2DM. However, since HbA1c was included both as a predictor and as part of the operational definition of the diabetes group, the findings should be interpreted with caution due to the risk of target leakage. Therefore, these results should be considered exploratory rather than evidence of clinically applicable predictive performance, and an independent external validation study should be conducted prior to clinical application.

## 1. Introduction

Type 2 diabetes mellitus (T2DM) is a progressive metabolic disorder characterized by insulin resistance and pancreatic β cell dysfunction, leading to chronic hyperglycemia. Today, diabetes represents one of the most important global public health challenges, with T2DM accounting for more than 90% of all diabetes cases worldwide [1]. The pathophysiology of the disease is multifactorial and involves not only insulin resistance and progressive β-cell dysfunction, but also increased hepatic glucose production, reduced incretin effect, and adipose tissue dysfunction [2]. Beyond its metabolic complexity, T2DM is increasingly recognized as a heterogeneous disorder with clinically distinct phenotypes that differ in disease progression and complication risk, further underscoring the need for more refined approaches to patient characterization [3].

Failure to identify T2DM in a timely manner may lead to microvascular complications such as retinopathy, nephropathy, and neuropathy, as well as macrovascular complications including cardiovascular disease and increased mortality risk. Therefore, improving the recognition and characterization of T2DM remains a clinically relevant objective [4]. In this context, approaches capable of extracting useful information from routinely collected clinical data may help support more efficient identification of individuals with diabetes-related metabolic abnormalities.

In conventional clinical practice, diabetes is diagnosed primarily on the basis of biochemical indicators such as fasting plasma glucose, oral glucose tolerance testing, and HbA1c levels. However, growing evidence suggests that routinely obtained laboratory variables, including hemogram parameters and lipid profiles, may also reflect the inflammatory, metabolic, and vascular disturbances associated with diabetes [5]. Supporting this view, a recent systematic review and meta analysis reported that patients with T2DM show significant alterations in total leukocyte count, several differential white blood cell counts, and red blood cell related indices compared with controls, suggesting that routine hematological markers may capture clinically relevant aspects of the diabetic state [6]. In addition, cohort-based machine learning research has shown that routinely measured hematological factors such as white blood cell count, red blood cell indices, and platelet-related variables may contribute meaningfully to T2DM classification models [7].

The digitalization of health data and the expansion of computational analytics have created new opportunities to evaluate multidimensional clinical datasets more effectively. Accordingly, machine learning (ML) algorithms have increasingly been explored in chronic disease research and clinical decision-support contexts because they can model complex relationships that may not be fully captured by conventional linear methods. Previous studies have reported the use of support vector machines and other classification algorithms in diabetes- and prediabetes-related settings [8], while more recent work has extended these approaches to large routine clinical and physical examination datasets. For example, interpretable ML models applied to physical examination records have demonstrated good diagnostic discrimination for T2DM and have identified clinically relevant indicators across age and sex subgroups [9]. Moreover, a recent review of machine learning applications using tabular data in T2DM concluded that most contemporary studies focus on current state diabetes identification and that tree-based methods are among the most frequently successful modeling approaches in this area [10].

Although machine learning has previously been applied to diabetes classification using routine clinical variables, the contribution of the present study lies in its exploratory comparative framework rather than in claiming a wholly new diagnostic algorithm. Specifically, this study simultaneously benchmarks eight supervised machine learning models under two analytically distinct conditions, with and without HbA1c, in order to make the influence of HbA1c-related target leakage explicit. In addition, the dataset combines anthropometric, biochemical, and complete blood count-derived hematological parameters that are routinely available in clinical practice, allowing the analysis to examine whether non-HbA1c variables retain discriminatory information after removal of the diagnostic-defining HbA1c variable. This design differentiates the study from prior work that mainly reports single modeling pipelines or focuses on broad routine examination datasets, because it directly evaluates the incremental and interpretable contribution of hematological and metabolic variables within a single-center clinical cohort. Therefore, the present study should be interpreted as a hypothesis-generating comparison of routinely available clinical features and model families, intended to inform future externally validated T2DM classification studies rather than to establish an immediately deployable clinical screening tool.

## 2. Materials and Methods

### 2.1. Study Design, Participants, and Data Collection

This single-center observational study was conducted at the Endocrinology Outpatient Clinic of Gaziantep Islam Science and Technology University, Faculty of Medicine, Ersin Arslan Training and Research Hospital. The study aimed to compare the performance of different machine learning algorithms for classifying individuals with and without type 2 diabetes mellitus (T2DM) using anthropometric, biochemical, and hematological parameters. A total of 160 individuals (95 females and 65 males) were included. Sample size estimation was performed using G*Power 3.1.7 with an alpha level of 0.05 and a statistical power of 0.80. The dataset included anthropometric measurements such as height, body weight, waist circumference, hip circumference, and waist-to-hip ratio, as well as biochemical markers including HbA1c, HDL, LDL, insulin, glucose, triglycerides, and complete blood count parameters. Inclusion criteria were age between 18 and 65 years, provision of written informed consent, and no history of abdominal surgery. The diabetes group was operationally defined using HbA1c ≥ 6.5%, whereas the comparison group consisted of individuals without T2DM according to the available clinical dataset. Individuals with suspected pregnancy, a history of cancer, chronic systemic inflammatory disease, or incomplete data were excluded from the study.

### 2.2. Machine Learning Framework and Data Preprocessing

Supervised learning modeling was used in this dataset to compare the classification performance of multiple algorithms. The aim of the study was to determine which model provided the best classification performance for distinguishing between individuals with and without T2DM. The dataset underwent preprocessing within each cross-validation training fold, including imputation of missing data and feature standardization where appropriate. All models were re-run without HbA1c and the results were evaluated. Instead of a single 80/20 split, stratified 5-fold cross-validation was applied. SMOTE was applied only to the training partitions within each fold to improve class balance and to avoid leakage from validation data. Important features were highlighted using SHAP analysis, and model reliability was enhanced using a calibration curve. Model performance was evaluated using metrics such as accuracy, precision, sensitivity and F1 score.

As HbA1c is among the candidate predictor variables and is also part of the operational definition of the diabetes group, the training results were first evaluated without HbA1c, and subsequently the model’s performance was analyzed with HbA1c integrated. The models obtained should be interpreted not as fully independent prediction models, but as classification models within this dataset.

In this study, hyperparameter optimization was not applied; instead, the models were trained using their existing/default parameter settings. Therefore, no parameter grid was used. To ensure reproducibility across all stochastic procedures, a random_state = 42 seed value was used for data partitioning, SMOTE, and applicable model training steps. Model evaluation was performed using the Stratified 5-Fold Cross-Validation method. Missing values were identified in some variables within the model dataset. Whilst the missing value rate was low, at around 0.63%, for most variables such as age, gender, income status, duration of diagnosis, kidney problems and loss of sensation, the highest missing value rate of 6.88% was observed in the educational status variable. Among the clinical variables, the missing value rate was 3.13% for APG, HbA1c and LDL, 3.75% for total cholesterol and HDL, and 1.25% for BMI and waist circumference. To address missing values, median imputation was applied within the training folds and then applied to the corresponding validation folds. This approach is robust against outliers and helps minimize leakage during model evaluation.

#### Machine Learning Models

The machine learning models to be used in this study are as follows:

Logistic Regression: One of the supervised learning models and a classification algorithm in artificial intelligence, logistic regression separates two classes into distinct groups. It is relatively straightforward to interpret, flexible, and achieves an optimum level by making classification more discriminative through imputation of missing values. As a discriminative method, logistic regression requires the use of discriminative methods in the dataset [11].

**Linear Discriminant Analysis (LDA):** The fundamental goal of linear analysis is to maximize the distinction between classes. It attempts to minimize the distance between classes and is a statistical and machine learning-based approach.

**Quadratic Discriminant Analysis (QDA):** Identifies the distribution that best separates classes from one another based on their distributions. It calculates a separate matrix for each class and utilizes probability distributions.

**Extra Tree Classifier (ETC):** It is one of the tree-based ensemble learning methods. It creates numerous decision trees within the network structure and combines their predictions.

**Decision Tree:** A Decision Tree is a supervised learning model that classifies or predicts data by splitting it into different branches. It makes decisions based on features and attempts to maintain predictions. It gathers all data at the root and splits it into branches, integrating it into classification algorithms of different structures [12,13,14].

**Random Forest:** Random Forest is a tree-based ensemble learning method that constructs multiple decision trees on bootstrapped samples and aggregates their predictions to improve robustness and reduce variance [14].

**K-Nearest Neighbors (KNN):** KNN looks at the nearest neighbors to classify a new observation. It calculates the distances to the nearest neighbors and assigns the observation to the most likely class accordingly.

**Gaussian Naive Bayes (GaussianNB):** This is a probability-based classification method based on Bayes’ theorem, which emphasizes that data is distributed proportionally.

### 2.3. Statistical Analysis

All analyses were performed using Python 3.12.13 (Google Colaboratory platform). Normal distribution was assessed using the Anderson–Darling test. Variables showing a normal distribution were presented as mean ± standard deviation and compared using the independent samples *t*-test; variables not showing a normal distribution were presented as median (minimum–maximum) and compared using the Mann–Whitney U test. Correlations between continuous variables were assessed using Pearson or Spearman correlation coefficients, as appropriate. Eight algorithms were used for machine learning analysis: Linear Discriminant Analysis (LDA), Quadratic Discriminant Analysis (QDA), Logistic Regression (LR), Extra Tree Classifier (ETC), Decision Tree (DT), Random Forest (RF), Gaussian Naive Bayes (GaussianNB) and k-Nearest Neighbours (k-NN)—and were evaluated using biochemical and haemogram variables. The dataset was evaluated using 5-fold stratified cross-validation rather than a single 80/20 split, and model performance was assessed using accuracy, sensitivity, specificity, F1 score and ROC-AUC. ROC curves were used for a descriptive evaluation of discriminatory performance. A calibration curve was used to demonstrate the accuracy of the probabilities and thereby enhance the model’s reliability. SHAP analysis was performed to interpret the effects of features on model predictions.

#### 2.3.1. Receiver Operating Characteristic (ROC) Analysis

ROC Curve: It illustrates how a binary classification model performs across a range of decision thresholds. Predicted probabilities are converted into class labels using a selected threshold, commonly 0.5.

If the threshold value decreases, more people are labeled as “positive”, and sensitivity increases.

If the threshold value increases, fewer people are labeled as “positive”, and specificity increases.

#### 2.3.2. Performance Metrics

Metrics Used

Accuracy: It measures the proportion of correctly classified observations among all observations. This metric indicates the overall classification accuracy of the model. The accuracy formula is shown in Equation (1).

(1) $$\mathrm{Accuracy}=\frac{\mathrm{TP}+\mathrm{TN}}{\mathrm{TP}+\mathrm{TN}+\mathrm{FP}+\mathrm{FN}}$$

Specificity (True Negative Rate): It shows what proportion of true negative results the model captures correctly.

(2) $$\mathrm{Specificity}=\frac{\mathrm{TN}}{\mathrm{TN}+\mathrm{FP}}$$

Sensitivity: It shows how well the model captures the true positive rate in its prediction results.

(3) $$\mathrm{Sensitivity}=\frac{\mathrm{TP}}{\mathrm{TP}+\mathrm{FN}}$$

F1-Score: It is the harmonic mean of Precision and Recall values. It is widely used in imbalanced and complex datasets.

(4) $$F1=\frac{2\;\times \;\mathrm{Precision}\;\times \;\mathrm{Recall}}{\mathrm{Precision}+\mathrm{Recall}}$$

## 3. Results

For each model classification, the dataset was analyzed under two conditions: excluding HbA1c and including HbA1c. Instead of a single 80/20 split, stratified 5-fold cross-validation was applied across the model-evaluation workflow. To address class imbalance while reducing leakage risk, SMOTE was applied only within the training partition of each cross-validation fold. The highest accuracy was achieved by the Random Forest model when HbA1c was included (98.0%). This indicates that ensemble tree-based models, particularly Random Forest, can achieve strong apparent classification performance in this single-center dataset. All metric results are presented in Table 1.

**Figure 1:**
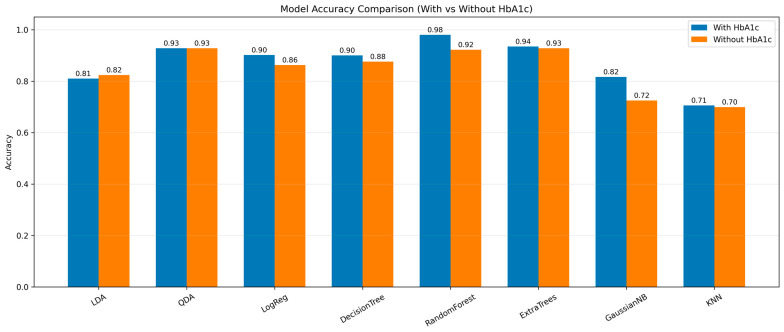
Model accuracy values.

The Extra Trees model achieved a success rate of 93.5%, whilst the Decision Tree model reached 90%, with these two results being close to each other. When the HbA1c analysis was not included, the models yielding the best results were Extra Trees and QDA, both achieving a similar success rate of 92.8%. Among all models, the lowest rate of 69.9% was obtained by the k-Nearest Neighbors (KNN) model. Table 1 shows the model performance metrics. Figure 1 shows the accuracy values of the models.

**Figure 2:**
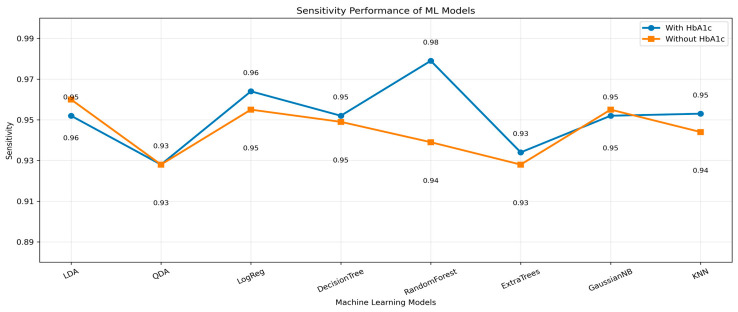
Sensitivity values of the models.

**Figure 3:**
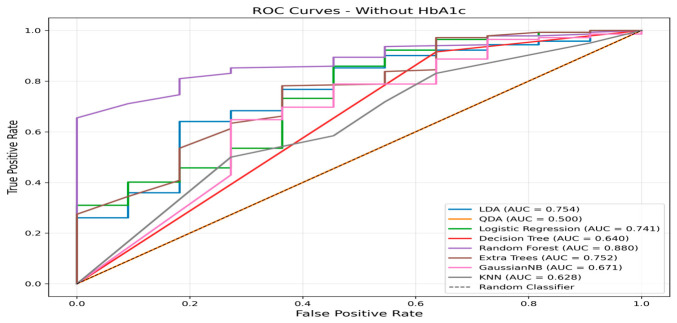
ROC curves without HbA1c.

**Figure 4:**
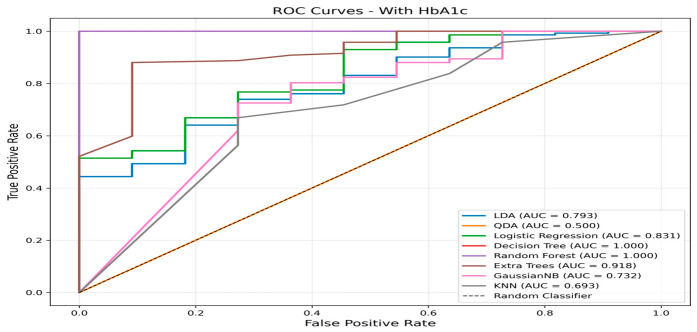
ROC curves with HbA1c.

In terms of sensitivity, when examining the model results, the highest success rate—97.9%—was achieved by the Random Forest model among those incorporating HbA1c. Success was achieved at a rate of 96.4% with Logistic Regression, 95.3% with KNN, 95.2% with GaussianNB and LDA, 93.4% with Extra Trees, and 92.8% with the QDA model. When examining the results of models that did not include HbA1c, the highest success rate of 96% was observed in the LDA model. When examining the model results in terms of F1 scores, the Random Forest model achieved the highest F1 score of 99.0% in the models including HbA1c. This was followed by Extra Trees at 96.6% and QDA at 96.3%; this indicates that all three models produced robust and consistent F1-score performance. Figure 2 shows the sensitivity values of the models with and without HbA1c. Figure 3 presents the ROC curves without HbA1c, whereas Figure 4 presents the ROC curves with HbA1c.

**Figure 5:**
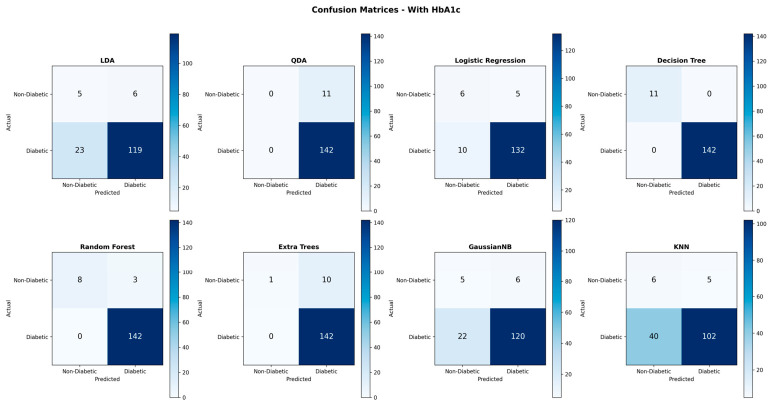
Confusion matrix with HbA1c.

**Figure 6:**
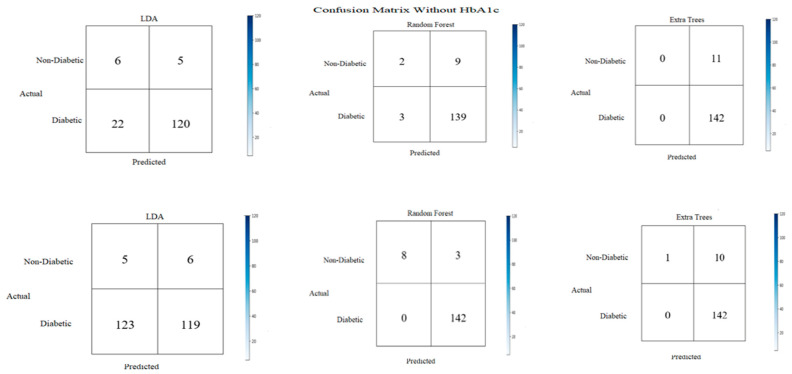
Confusion matrix without HbA1c.

On the ROC curve graph, the x-axis represents the false positive rate (1 − specificity), whilst the y-axis represents the sensitivity. The threshold value on the ROC curve starts at 1 and is gradually reduced towards 0. Metrics are calculated for each threshold value and a ROC curve is plotted. The area under the ROC curve is referred to as the AUC. As the AUC value approaches 1, the model’s performance improves. Curves on the ROC plot that lie closer to the top-left corner indicate better discriminatory performance. ROC curves demonstrated positive discriminatory performance for tree-based models, particularly Random Forest and Logistic Regression. The corresponding ROC-AUC values are reported in Table 1; nevertheless, given the single-center design and the limited sample size, these results should be interpreted as exploratory rather than as definitive evidence of optimal diabetes classification performance. Confusion matrices were generally consistent with overall performance metrics; whilst the Random Forest model showed the highest proportion of correctly classified observations, QDA exhibited a relatively higher number of misclassifications. Figure 4 shows the ROC curves without HbA1c. Figure 5 and Figure 6 show the confusion matrices for the machine learning models evaluated with and without HbA1c, respectively. Two different confusion matrix plots are provided. The confusion matrices are divided into two categories: one including HbA1c and one excluding HbA1c. Upon examining the plots, it is clearly evident that the Random Forest and Extra Trees models classify the data more effectively than the other models.

When examining the confusion matrix plots, we can see that two different models are presented: one with HbA1c included and one without. It is evident that including HbA1c significantly improves the model’s accuracy. Furthermore, even when HbA1c is not included, the model achieves significant success in the confusion matrix plots by minimizing the number of incorrect classifications. The confusion matrix excluding HbA1c is shown in Figure 6.

**Figure 7:**
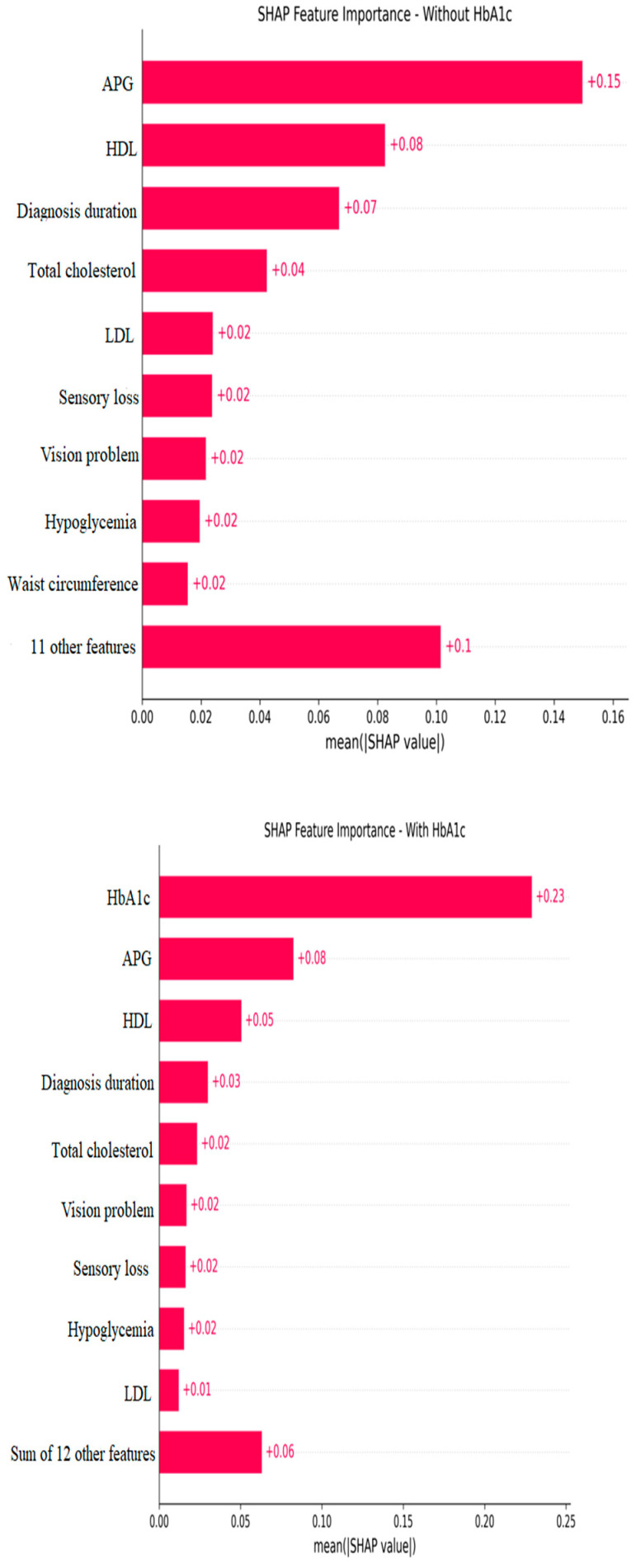
SHAP feature importance without HbA1c and with HbA1c.

In the model estimates, it is observed that the most influential variable without HbA1c is APG, followed by HDL and the duration of diagnosis. When HbA1c is included in the model, it is observed that the HbA1c variable yields the highest value. This is because the HbA1c variable reflects long-term blood levels and is a clinically significant marker in the diagnosis of diabetes (Figure 7).

**Figure 8:**
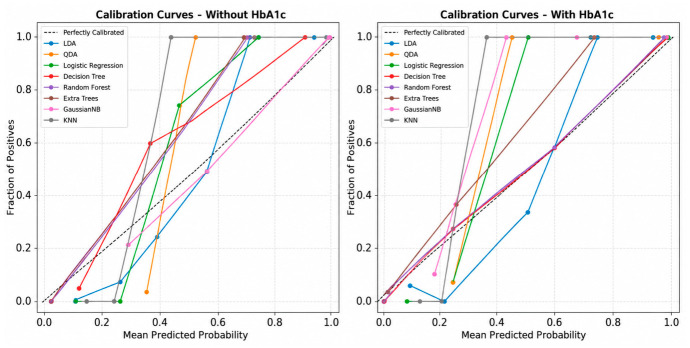
Calibration curves without HbA1c and with HbA1c.

The calibration curves show the agreement between predicted probabilities and observed outcomes. Curves closer to the diagonal reference line indicate better calibration. Figure 8 presents calibration curves for models evaluated with and without HbA1c. Figure 8 shows calibration curves with and without HbA1c.

## 4. Discussion

In this study, it was demonstrated that machine learning models based on biochemical and hemogram parameters can predict the diagnosis of type 2 diabetes mellitus (T2DM) with high accuracy rates. In particular, the superior performance of the Random Forest (98.0%), Extra Trees (93.5%), and Decision Tree (90.0%) models indicates that non-linear and ensemble methods are effective in the classification of metabolic diseases.

Insulin resistance, β cell dysfunction, and systemic inflammation are all part of the complicated pathophysiology of type 2 diabetes [1,2]. Non-linear interactions between biochemical and hematological indicators are a result of this multifactorial structure. As a result, models with linear assumptions (like LDA) typically perform worse, while ensemble algorithms and decision trees are anticipated to yield better outcomes. According to Breiman’s research on the Random Forest algorithm, ensemble approaches lower variance and produce more reliable and accurate results [15]. Our study’s results are in line with this theoretical paradigm.

The pathophysiology of type 2 diabetes is a multifactorial process. Anthropometric parameters provide information about insulin resistance, particularly by indicating abdominal obesity and visceral fat accumulation. Increased BMI is associated with insulin resistance, especially as an indicator of visceral fat accumulation. TNF-α, IL-6, and free fatty acids released from adipose tissue disrupt insulin signaling pathways, reducing glucose utilization. This increases glucose production in the liver while decreasing glucose uptake in peripheral tissues. Obesity leads to insulin resistance, which in turn causes hyperglycemia [16]. Abdominal obesity is a stronger risk indicator for type 2 diabetes than BMI. Increased inflammatory cytokine production in visceral fat tissue leads to hepatic insulin resistance, hyperinsulinemia, and dyslipidemia [17].

Biochemical parameters reflect glycemic control, lipid metabolism, and inflammation levels. HbA1c is a parameter that shows the average glucose level over the last 2–3 months and is associated with microvascular complications, oxidative stress, and endothelial damage [18]. Elevated HOMA-IR, used to assess insulin resistance, indicates decreased glucose utilization in muscle tissue, increased hepatic glucose production, and increased β-cell load. In type 2 diabetes, increased triglycerides, decreased HDL, and increased LDL are observed. Insulin resistance disrupts lipoprotein metabolism, leading to atherogenic dyslipidemia. This increases the risk of cardiovascular disease [17].

Hematological parameters can be used to assess chronic inflammation and vascular effects in diabetes. Inflammation underlies hematological changes in T2DM. Pro-inflammatory cytokines can increase leukocyte production by affecting bone marrow activity. Elevated WBC levels are indicative of systemic inflammation. In type 2 diabetes, leukocyte levels may rise due to chronic inflammation. High WBC is also associated with insulin resistance [16]. Some studies have shown that increases in hemoglobin (Hb), hematocrit (Hct), and erythrocyte count are associated with insulin resistance and β-cell dysfunction. Increased oxidative stress, inflammation, and impaired microvascular circulation may be the cause. Chronic hyperglycemia causes glycation of erythrocyte proteins and reduces erythrocyte deformability. This impairs microcirculation and contributes to diabetic complications [19]. The combined assessment of these parameters is important in predicting both the risk of developing diabetes and its complications.

In the literature, various machine learning algorithms have been successfully applied in studies on diabetes prediction. Studies conducted using the Pima Indians Diabetes dataset, developed by Smith and colleagues, have demonstrated that logistic regression and decision trees are effective in the classification of diabetes [20]. In a more recent study, Zou and colleagues reported that when machine learning models are used in combination with clinical parameters, they demonstrate higher performance in predicting diabetes risk compared to traditional statistical methods [21]. Similarly, in their systematic review, Kavakiotis and colleagues emphasized that ensemble and deep learning methods yield superior results in the prediction of chronic diseases [22].

The findings should therefore be positioned within an already active literature on machine learning-based diabetes classification. The present study does not suggest that applying machine learning to routine clinical variables is itself novel. Rather, its added value is threefold: first, it provides a side-by-side comparison of several linear, probabilistic, distance-based, and tree-based classifiers in the same clinical dataset; second, it explicitly contrasts model performance with and without HbA1c to address the diagnostic-threshold leakage concern; and third, it evaluates whether routinely measured hematological markers and metabolic variables contribute to classification when HbA1c is excluded. This distinction is important because previous work has shown that tree-based methods often perform well in tabular T2DM datasets, while hematological parameters may also reflect diabetes-related inflammatory and metabolic alterations [7,9,10]. Accordingly, the current results mainly advance the field by clarifying how much apparent performance depends on HbA1c and by identifying APG, HDL, and diagnosis duration as influential non-HbA1c features in this single-center exploratory dataset.

The ROC curve analysis demonstrated that all machine learning models achieved high discriminative performance, particularly the tree-based algorithms such as the Extra Trees, Random Forest, and Decision Tree models. The superior AUC values observed in these models suggest that non-linear relationships and complex feature interactions may play an important role in distinguishing metabolic patterns associated with diabetes. Previous studies have similarly reported that ensemble and tree-based machine learning approaches often outperform conventional statistical models in clinical prediction tasks due to their ability to capture complex multidimensional relationships within biomedical datasets. In addition, the high true positive rates achieved at relatively low false positive rates indicate that the developed models may have potential clinical utility for classification purposes [23].

ROC curves and AUC values are important metrics for evaluating model performance. Bradley stated that the AUC is a reliable indicator of classification performance. In our study, the high discriminative ability of tree-based models on the ROC curves indicates that these models provide stable performance across different threshold values [24]. The combined evaluation of Precision–Recall and ROC curves provides more meaningful results, particularly in imbalanced datasets [25].

HbA1c is a well established biomarker in the diagnosis of diabetes [26]. In the present study, HbA1c was included among the predictor variables, while HbA1c ≥ 6.5% was also used as part of the operational definition for the diabetes group. Therefore, the observed model performance should be interpreted with caution, as part of the discriminative performance may reflect the inclusion of a diagnostic-defining variable within the predictor set. Although hematological and biochemical parameters may jointly improve model discrimination, this design feature may have inflated performance estimates, particularly for models that strongly exploit dominant predictors. Future studies should evaluate model performance both with and without HbA1c, and ideally in externally validated datasets, to better determine the independent contribution of non-HbA1c variables.

The potential of machine learning-based decision-support systems in clinical practice is steadily increasing [4,5]. However, large-scale, multicenter studies with external validation are required to ensure the reliability of these systems. Since our study was conducted at a single center with a relatively limited sample size, its generalizability is restricted. Future studies with larger datasets and prospective validation are needed to support the clinical integration of these models.

In conclusion, machine learning models based on biochemical and hemogram parameters showed favorable apparent classification performance for T2DM in this single-center dataset. Tree-based and ensemble methods yielded the most promising results; however, the findings remain preliminary and should not be considered ready for clinical decision-support use without external validation and further model refinement.

This study has several limitations that should be considered when interpreting the findings. First, the study was conducted at a single center with a relatively small sample size following data cleaning (*n* = 153), which may limit the generalizability of the results to wider populations. To improve the stability of the performance estimates, stratified 5-fold cross-validation was used instead of a single 80/20 split. Therefore, larger multicenter studies are required to validate the robustness and reproducibility of the proposed models. Second, no external validation assessment was conducted using an independent cohort. The absence of external validation limits the ability to evaluate the true generalization performance of the machine learning models.

Whilst HbA1c was included as one of the predictor variables, the value HbA1c ≥ 6.5% was also used as part of the definition of the diabetes group. This situation creates a risk of target leakage and may have led to an over-optimistic estimation of classification performance. Therefore, the findings should be interpreted as exploratory classification results within this dataset rather than as definitive evidence of clinically applicable predictive performance. As the study was conducted using two different parameters—with and without HbA1c—we sought to make the analysis of the results more robust by examining how the model behaved under different decision structures. In future analyses, models employing different structures or alternative outcome definitions should be evaluated to better assess the incremental value of the remaining anthropometric, biochemical, and hematological variables. No hyperparameter optimization was performed in the present study; therefore, future work should also examine whether model tuning improves generalizability without increasing the risk of overfitting.

## 5. Conclusions

In conclusion, routine clinical laboratory and anthropometric variables showed favorable apparent performance for classifying individuals with and without T2DM in this single-center exploratory dataset. The strongest performance was observed for tree-based and ensemble models, particularly Random Forest and Extra Trees; however, the comparison of models with and without HbA1c indicated that part of the performance advantage was likely attributable to the inclusion of a diagnostic-defining biomarker. The main contribution of this study is therefore the transparent comparison of multiple model families, the explicit HbA1c leakage sensitivity analysis, and the assessment of whether hematological and metabolic variables retain discriminatory information beyond HbA1c. These findings should be regarded as preliminary and hypothesis-generating rather than immediately generalizable to clinical practice. Future multicenter studies using independent datasets, external validation, and refined modeling strategies are required before these approaches can be recommended for clinical screening or decision-support applications.

## Figures and Tables

**Table 1:** Model performance metrics with HbA1c included and without HbA1c.

Model	Accuracy (with HbA1c)	Accuracy (Without HbA1c)	Sensitivity (with HbA1c)	Sensitivity (Without HbA1c)	Specificity (with HbA1c)	Specificity (Without HbA1c)	F1 (with HbA1c)	F1 (Without HbA1c)	AUC (with HbA1c)	AUC (Without HbA1c)
LDA	0.810	0.824	0.952	0.960	0.838	0.845	0.891	0.899	0.793	0.754
QDA	0.928	0.928	0.928	0.928	0.917	0.886	0.963	0.963	0.500	0.500
Logistic Regression	0.902	0.863	0.964	0.955	0.930	0.894	0.946	0.924	0.831	0.741
Decision Tree	0.900	0.876	0.952	0.949	0.981	0.915	0.935	0.932	1.00	0.640
Random Forest	0.980	0.922	0.979	0.939	0.988	0.979	0.990	0.959	1.00	0.880
Extra Trees	0.935	0.928	0.934	0.928	0.974	0.962	0.966	0.963	0.918	0.752
GaussianNB	0.817	0.725	0.952	0.955	0.845	0.739	0.896	0.833	0.732	0.671
KNN	0.706	0.699	0.953	0.944	0.718	0.718	0.819	0.816	0.693	0.628

## Data Availability

The datasets generated and/or analyzed during the current study are not publicly available due to privacy restrictions but are available from the corresponding author on reasonable request.

## Abbreviations

The following abbreviations are used in this manuscript:

T2DM	Type 2 Diabetes Mellitus
ML	Machine Learning
FPG	Fasting Plasma Glucose
HbA1c	Glycated Hemoglobin
HDL	High-Density Lipoprotein
SHAP	SHapley Additive exPlanations
SMOTE	Synthetic Minority Oversampling Technique
BMI	Body Mass Index
TP	True Positive
TN	True Negative
FN	False Negative
AUC	Area Under the Receiver Operating Characteristic Curve
